# The Book World of Medicine and Science

**Published:** 1896-07-18

**Authors:** 


### The Book World of Medicine and Science.

Southall's Organic Materia Medica (fifth and enlarged edition). By John Barclay, B.Sc. Lond. (London: J. and A. Churchill. 1896. Price 6s.)

The fourth edition of this work was printed in 1887, and during the last few years it has been out of print. The method of arrangement has been altered; drugs of vegetable origin no longer are classified according to the natural order to which they belong, but as far as possible they have been arranged according to their forms and physical characters. Notes on the chemical properties of the various drugs have been added, and, to a certain extent, information as regards the therapeutic action. All the drugs comprised in the British Pharmacopœia are dealt with, and in addition certain others which have found place in such popular works as "Squire's Companion" and "Martindale's Extra Pharmacœpia." This new addition, which has been carefully and systematically drawn up, should be specially useful to teachers in materia media, since it includes in a succinct form recent information which can only otherwise be obtained by a reference to a number of different works and periodicals which treat of such diverse subjects as botany, chemistry, pharmacy, and therapeutics.

Surgical Diseases of the Ovaries and Fallopian Tubes, including Tubal Pregnancy. By J. Bland Sutton, F.R.C.S. (London: Cassell and Co. New and enlarged edition. Illustrated. 1896. Price 21s.)

We would at once express our admiration of this work. Too often is it the case that much reading makes a man diffuse, and his writings a dull record of other people's opinions; but here we have the results of large experience and personal investigation, combined with a wide survey of the literature of the subject; the author not only expresses his own opinions, but gives his reasons; and all this he does in such a manner as to produce a book which, besides being authoritative, is eminently readable. The first 190 pages are occupied with a description of diseases of the ovaries and their treatment. The fibromata, myomata, sarcomata, and carcinomata are dismissed in a comparatively small space, but the main tumours of the ovaries and their appendages, the adenomata, dermoids, and cysts, are very fully discussed. The examples given of ovarian dermoids, and the plates by which these chapters are illustrated, are of very great interest. The author is strongly opposed to the view held by some that these dermoids sometimes become malignant. He says "cases have crept into literature in which surgeons believed recurrence had taken place, but it has never been demonstrated by actual inspection of the parts in the course of an operation or a *post-mortem* examination." Many of the cases which have seemed to some to indicate infection of the peritoneum seem to the author to be cases of epithelial infection analogous to what are described as implantation cysts. The chapters dealing with the secondary changes in ovarian tumours—inflammation and suppuration, axial rotation, and rupture of the cyst—are full of interest to the practical surgeon, as also are those dealing with other complications, such as pregnancy. A very careful description is given of the results of inflammation of the Fallopian tubes, and of the rare instances in which neoplasms form therein. The author has no hesitation in advising removal where there is either pyosalpinx, hydrosalpinx, tubercular salpingitis, or ovarian abscess, which produce so much pain as to cause the patient to lead the life of a chronic invalid. "To dilate the cervical canal and scrape the interior of the uterus, or tap a pyosalpinx through the vagina, or attempt to disperse pus by electricity, by moor-baths, or by mild purgatives, are modes of treatment which can only be described as ridiculous, and in many cases they are highly dangerous." He is, however, quite opposed to the removal of healthy, or so-called cystic ovaries for the relfef of neuroses. Space forbids our doing more than mention the parts of the book devoted to the consideration of tubal pregnancy and operative methods. It must be said, however, especially in regard to the former, that they are admirable expositions of what is known on the subject and of the author's practice. The book is well printed, admirably illustrated, interesting to read, and, besides giving an impartial account of the observations of others, is a personal record of good work.

